# Tachometer for Reverse Cholesterol Transport?

**DOI:** 10.1161/JAHA.112.003723

**Published:** 2012-08-24

**Authors:** Arnold Eckardstein

**Affiliations:** Institute of Clinical Chemistry, University Hospital Zürich and Zürich Center for Integrative Human Physiology, University of Zürich, Zürich, Switzerland

**Keywords:** cholesterol efflux, high-density lipoprotein, reverse cholesterol transport

Many clinical and epidemiological studies, as well as meta-analyses thereof, have shown the inverse relationship of high-density lipoprotein cholesterol (HDL-C) and apoliopoprotein (apo) A-I plasma levels with the risk of coronary artery disease.^1^ HDL particles and their protein and lipid components exert diverse antioxidative, antiinflammatory, antithrombotic, and cytoprotective effects in vitro and in vivo, which suggests a direct antiatherogenic role of HDL. Moreover, the development of atherosclerotic lesions could be decreased or even reverted in several animal models by transgenic overexpression or exogenous application of apoA-I, the most abundant protein of HDL. Infusion of artificially reconstituted HDL was found to improve endothelial dysfunction and reduce atherosclerotic plaque volume of patients with coronary artery disease.^2–3^

For low-density lipoprotein cholesterol (LDL-C) and high blood pressure, this type of epidemiological and biological evidence has been successfully translated into drugs that lower cardiovasuclar risk. To date, however, it has proved difficult to successfully reduce cardiovascular risk with HDL-C–increasing or –modulating drugs, such as fibrates, niacin, inhibitors of cholesteryl ester transferprotein (CETP) and infusions of reconstituted HDL.^2,4^ Moreover, in several inborn errors of human HDL metabolism and in genetic mouse models with altered HDL metabolism, changes in HDL-C levels were not associated with the opposite changes in cardiovascular risk and atherosclerotic plaque load that were expected from epidemiological studies.^2–3,5^ Because of these controversial data, the pathogenic role of HDL and its suitability as a therapeutic target have been increasingly questioned. In this controversy, it is important to note that previous intervention and genetic studies targeted LDL-C and HDL-C—that is, the cholesterol measured by clinical laboratories in LDL and HDL, respectively. In contrast to the proatherogenic and disease-causing cholesterol in LDL (ie, LDL-C), which after internalization turns macrophages of the arterial intima into proinflammatory foam cells, the cholesterol in HDL (ie, HDL-C) does not exert any of the potentially antiatherogenic activities of HDL. In contrast to LDL-C, HDL-C is only a nonfunctional surrogate marker for estimating HDL particle number and size without deciphering the heterogeneous composition and, hence, functionality of HDL.^2–3^ Previous proteomic and lipidomic studies revealed that HDL particles carry >80 different proteins and hundreds of lipid species.^2–3^ Most recently, even microRNAs were found to be transported by HDL.^6^ Many of these molecules are not passive cargo but are biologically active and contribute to the pleiotropic and potentially antiatherogenic properties of HDL. Interestingly, HDL of patients with various inflammatory diseases, including diabetes mellitus type 2, coronary artery disease, and rheumatoid arthritis, was found to have lost potentially vasoprotective functions, such as stimulation of cholesterol efflux^7^ and endothelium-dependent vasodilation,^4,8^ or even to have acquired adverse endothelial dysfunctions^4,9^ beyond what is measured with plasma concentrations of HDL-C or apoA-I. Therefore, a great clinical need exists for biomarkers that reflect the functionality of HDL more directly and thus with higher sensitivity and specificity than HDL-C or apoA-I. Unfortunately, however, it is not known which of the many potentially antiatherogenic functions and hence molecules of HDL are the most important ones and the best candidates to be exploited for the development of functional HDL biomarkers. While searching for such biomarkers, many laboratories have developed bioassays, most of which are cell based and work in vitro.

In this issue of the *Journal of the American Heart Association*, Scott Turner et al^10^ report a novel and noninvasive method that aims to measure a classic, putatively antiatherogenic function of HDL in humans and in vivo—namely, reverse cholesterol transport (RCT). RCT describes the transport of excess cholesterol from peripheral cells to the liver for excretion into the bile, either directly or indirectly after conversion into bile acids, or to steroidogenic organs for hormone production (Figure 1).^11^ Turner and colleagues infused [2,3-^13^C_2_] cholesterol into healthy volunteers^10^ and measured and modeled 3 important components of RCT: efflux and esterification of cholesterol in plasma, as well as fecal sterol excretion. As discussed by the authors, their study protocol improves on previous ones by the use of a stable isotope of cholesterol, which makes it applicable to humans without any ethical limitations. The long-term infusion together with the applied modeling strategy allow differentiation of unproductive equilibration of the tracer with natural cholesterol in the plasma membranes and thus allow the measurement of net efflux. An additional strength of this protocol is the integration of several RCT steps, which otherwise would have been recorded by single surrogate in vitro assays, into one in vivo setting. The protocol's applicability to clinical studies is also a strength. The major limitation of this protocol is that it follows the model of RCT that was introduced nearly 50 years ago by Glomset and Wright,^12^ which by current standards is oversimplified. Since its introduction, the model has been refined extensively by the discovery of several rate-limiting genes and by the metabolic characterization of patients and genetic mouse models that lack these limiting factors.^11^

1. In the original concept of Glomset and Wright,^12^ HDL induces net efflux of cellular cholesterol because its extracellular esterification by lecithin:cholesterol acyltransferase (LCAT) prevents rediffusion and hence equilibration of cholesterol between cell membranes and lipoproteins. Turner et al^10^ suggest that the erythrocyte–plasma exchange flux, which according to their data amounts to about two thirds of total cholesterol efflux, could be a surrogate marker of this process. According to the model of Glomset and Wright, LCAT activity is a driving force, especially for aqueous diffusion of cholesterol, so it will be interesting to see in future studies how LCAT deficiency affects this process and whether the concentration or activity of LCAT correlates with red blood cell cholesterol efflux. In this regard, it is interesting to note that LCAT concentrations are not associated with coronary artery disease risk, at least if taken per se, and that LCAT is still under consideration as a target for antiatherosclerotic therapy.^13–14^
2. To date, in addition to aqueous diffusion, which is facilitated by tethering of HDL to scavenger receptor BI, 2 ATP-binding cassette transporters, ABCA1 and ABCG1, are known to mediate active export of excess cholesterol to lipid-poor apoA-I that is preβ1-HDL and lipid-enriched α-HDL, respectively (Figure 1).^11,15^ Both transporters are integrated into both positive feed-forward and negative feed-back regulation loops. In situations of excess cellular cholesterol, the nuclear liver X receptors LXR-α and LXR-β induce the transcription of ABCA1 and ABCG1 and thus cholesterol efflux.^15^ Cells lacking cholesterol produce several microRNAs (eg, miR33a, miR33b) that decrease the production of ABCA1 and thus cholesterol efflux.^16^ Consequently, cholesterol efflux activity is determined by the extracellular concentration and composition of HDL particles as well as by the activity of cellular ABC transporters. Many laboratories worldwide have developed variations of in vitro bioassays in which either the cellular cholesterol efflux activity of fibroblasts or monocytes or the cholesterol acceptor capacity of HDL, total serum or plasma, or apoB-depleted serum or plasma was investigated. Both monocyte-derived macrophages and fibroblasts of patients with functionally relevant mutations in ABCA1 show gene dosage–dependent decreases in cellular cholesterol efflux capacity.^17^ Decreased cholesterol efflux also was seen in monocytes of patients with diabetes mellitus.^18^ Many different protocols have been reported for the measurement of cholesterol efflux capacity of HDL; the protocols differ by the cell type used as the donor of cholesterol (mostly fibroblasts or different kinds of macrophages); by the use of HDL, serum, or plasma with or without depletion of apoB-containing lipoproteins; and by efflux times (from 1 minute up to several hours).^7,19–23^ Interestingly, total and apoB-depleted plasmas of patients with virtually complete HDL deficiency show strongly reduced but still considerable residual cholesterol acceptor properties, which have been explained by passive efflux to albumin and LDL as well as by active efflux to minor HDL subfractions like preβ1-HDL.^20^ After its prominent publication, the protocol by Khera and colleagues^7^ incubating apoB-depleted serum with a mouse cell line with induced ABCA1 expression became most popular and a kind of “gold standard.” Nevertheless, it must be emphasized that the use of apoB-depleted serum neglects the contribution of LDL and very-low-density lipoprotein to cholesterol efflux capacity^21–22^ as well as the conversion of HDL subclasses and the proteolytic degradation of preβ1-HDL during coagulation of serum.^23–24^ Moreover, during 4 hours of incubation, cellular receptor interactions as well as influx of serum components (eg, cytokines, free fatty acids) could modulate the activity of ABCA1, ABCG1, and other cellular components of the cholesterol efflux machinery.^15^ These confounders could strongly influence cholesterol efflux capacity of serum independently of HDL composition and functionality. With this background, it will be interesting to see how total cholesterol efflux as measured by Turner et al^10^ correlates with the various measures of cholesterol efflux capacity of cells and plasma/serum with or without apoB depletion.
3. On the basis of the model of Glomset and Wright, many people still erroneously believe that HDL-C reflects the amount of cholesterol that is released from peripheral cells and is transported by HDL back to the liver. However, in mice, both transplantation of ABCA1-deficient bone marrow and targeted knockout of ABCA1 in macrophages clearly demonstrated that cholesterol efflux from macrophages affects the extent of atherosclerosis but not levels of HDL-C.^25–26^ At first sight in agreement with this, Turner and colleagues did not observe any significant correlation between total cholesterol efflux and HDL-C levels. However, this lack of correlation is also surprising, because according to the existing dogma, the concentration or number of HDL particles, which also is reflected by HDL-C, should be an important determinant of cholesterol efflux capacity of plasma. However, as Turner et al admit, the number of investigated subjects was too small for definite conclusions. We have to await the results of future and larger studies to see this and other correlations—for example, with HDL subclasses and functional HDL biomarkers, as well as with apoB-containing lipoproteins, the concentrations and turnover rates of which were identified as determinants of plasma cholesterol efflux capacity in vitro.^21–22,27^
4. Originally, RCT was believed to involve all nonhepatic and nonsteroidogenic cells. This concept first was challenged by Dietschy and colleagues,^28^ who showed that HDL-deficient mice with knockouts of ABCA1 or apoA-I excrete normal amounts of sterols with their feces and show no disturbances in whole-body cholesterol homeostasis. In both patients and mice lacking functional ABCA1, cholesterol accumulation occurs only in macrophages. This observation induced Rader, Rothblat, and colleagues to introduce a novel experimental animal model of macrophage-specific RCT. Peritoneal or bone marrow–derived macrophages are labeled with radioactive cholesterol and then installed into the peritoneum of recipient mice for the measurement of radioactive cholesterol taken up into plasma or liver as well as excreted into feces.^29^ In this model, interferences with either the cellular cholesterol efflux machinery (eg, by knockout of ABCA1) or the concentration and composition of HDL in plasma (eg, by knockout or overexpression of apoA-I) led to expected alterations in RCT. Compared to alterations in HDL-C, these alterations in RCT also showed stronger correlations with changes in atherosclerosis in different animal models of disturbed HDL metabolism.^29^ Consequently, many investigators have used this model or modifications thereof for testing the effect of diseases and therapeutic interventions on RCT.^29^ Unfortunately, and as an admitted limitation, the method described by Turner et al does not allow discrimination of macrophage-specific RCT from overall RCT.
5. In the protocol of Turner et al,^10^ large proportions of the infused isotopic cholesterol tracer equilibrate with endogenous nonisotopic cholesterol of erythrocyte membranes. Physiologically, the majority of cholesterol efflux takes place in the extravascular compartment, which is separated from the blood stream by endothelial barriers.^30^ Therefore, the protocol red blood cell cholesterol efflux relative to total cholesterol efflux is probably overrepresented in the model of Turner et al.^10^ It will be interesting to see in follow-up studies the metabolism of cholesterol in this compartment—for example, in the lymph.
6. After efflux, binding, and esterification, cholesterol is transported by several pathways to the liver. In humans, cholesteryl ester transfer protein transfers most of the cholesteryl esters to apoB-containing lipoproteins, which then are removed by the LDL receptor pathway.^11^ RCT thus involves both formation and catabolism of LDL. Consequently, both deficiency and pharmacological inhibition of cholesteryl ester transfer protein increase HDL-C and decrease LDL-C.^2,4^ Significant proportions of HDL-C also seem to be removed by selective uptake through scavenger receptor BI into the liver and steroidogenic organs, because mutation and knockout of scavenger receptor BI cause increases in HDL-C in humans and mice, respectively.^31^ In addition to these 2 well-characterized pathways of concluding RCT, at least 2 additional ones are emerging. One involves the holoparticle uptake of HDL through an as yet unidentified endocytic receptor that is activated by binding of apoA-I to ectopic β-ATPase and subsequent activation of purinergic receptors.^32^ Another involves the liver-independent transintestinal excretion of cholesterol, which appears to be independent of HDL (Figure 1).^33^ The fecal sterol excretion recorded by Turner and colleagues seems to integrate these different pathways of sterol elimination from the body. However, it is still unknown whether the mode of delivery and excretion of cholesterol is of any relevance for the antiatherogenicity of RCT.

**Figure 1.**
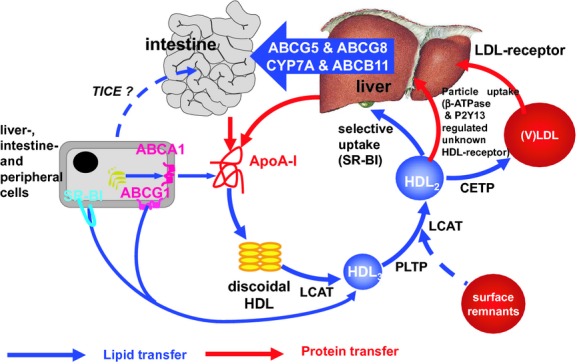
Key steps of RCT. HDL metabolism is a multistep process that involves the secretion of lipid-free apolipoproteins by the liver or intestine; acquisition of phospholipids and cholesterol from cells via ATP-binding cassette transporters (ABC) A1 and G1, as well as via scavenger receptor BI (SR-BI); maturation by LCAT-mediated cholesterol esterification and phospholipid transfer protein (PLTP)–mediated particle fusion; and final delivery of lipids to the liver, either directly via SR-BI or indirectly via cholesteryl ester transfer protein (CETP)–mediated transfer to LDL. Moreover, at least one as yet unidentified receptor stimulated by activation of the ektoATPase/P2Y13-axis mediates holoparticle uptake of HDL. The liver secretes cholesterol into the bile, either directly via ATP cassette transporters G5 and G8 or indirectly after oxidation to bile acid secretion via ATP cassette transporter B11. In addition to this classic hepatobiliary RCT mediated by HDL, there is increasing evidence for direct and HDL-independent transintestinal cholesterol excretion (TICE).

In conclusion, the newly described method of Turner and colleagues for the measurement of RCT offers many opportunities for the in vivo validation of (patho)physiological concepts in humans that previously emerged from experiments performed in vitro or in rodents. Still, the setting has limitations that should be kept in mind for interpretation and extrapolation of data obtained with this method.

## References

[1] Di Angelantonio E Sarwar N Perry P Kaptoge S Ray KK Thompson A Wood AM Lewington S Sattar N Packard CJ Collins R Thompson SG Danesh J Major lipids, apolipoproteins, and risk of vascular disease. JAMA. 2009;302:1993-2000 19903920 10.1001/jama.2009.1619 PMC3284229

[2] von Eckardstein A Implications of torcetrapib failure for the future of HDL therapy: is HDL-cholesterol the right target?. Expert Rev Cardiovasc Ther. 2010;8:345-358 20222814 10.1586/erc.10.6

[3] Besler C Lüscher TF Landmesser U Molecular mechanisms of vascular effects of high-density lipoprotein: alterations in cardiovascular disease. EMBO Mol Med. 2012;4:251-268 22431312 10.1002/emmm.201200224 PMC3376856

[4] Landmesser U von Eckardstein A Kastelein J Deanfield J Lüscher TF Increasing high-density lipoprotein cholesterol by cholesteryl ester transfer protein-inhibition: a rocky road and lessons learned? The early demise of the dal-HEART programme. Eur Heart J. 2012;33:1712-1715 22696435 10.1093/eurheartj/ehs182

[5] Voight BF Peloso GM Orho-Melander M Frikke-Schmidt R Barbalic M Jensen MK Hindy G Hólm H Ding EL Johnson T Schunkert H Samani NJ Clarke R Hopewell JC Thompson JF Li M Thorleifsson G Newton-Cheh C Musunuru K Pirruccello JP Saleheen D Chen L Stewart AF Schillert A Thorsteinsdottir U Thorgeirsson G Anand S Engert JC Morgan T Spertus J Stoll M Berger K Martinelli N Girelli D McKeown PP Patterson CC Epstein SE Devaney J Burnett MS Mooser V Ripatti S Surakka I Nieminen MS Sinisalo J Lokki ML Perola M Havulinna A de Faire U Gigante B Ingelsson E Zeller T Wild P de Bakker PI Klungel OH Maitland-van der Zee AH Peters BJ de Boer A Grobbee DE Kamphuisen PW Deneer VH Elbers CC Onland-Moret NC Hofker MH Wijmenga C Verschuren WM Boer JM van der Schouw YT Rasheed A Frossard P Demissie S Willer C Do R Ordovas JM Abecasis GR Boehnke M Mohlke KL Daly MJ Guiducci C Burtt NP Surti A Gonzalez E Purcell S Gabriel S Marrugat J Peden J Erdmann J Diemert P Willenborg C König IR Fischer M Hengstenberg C Ziegler A Buysschaert I Lambrechts D Van de Werf F Fox KA El Mokhtari NE Rubin D Schrezenmeir J Schreiber S Schäfer A Danesh J Blankenberg S Roberts R McPherson R Watkins H Hall AS Overvad K Rimm E Boerwinkle E Tybjaerg-Hansen A Cupples LA Reilly MP Melander O Mannucci PM Ardissino D Siscovick D Elosua R Stefansson K O'Donnell CJ Salomaa V Rader DJ Peltonen L Schwartz SM Altshuler D Kathiresan S Plasma HDL cholesterol and risk of myocardial infarction: a mendelian randomisation study. Lancet Published online before print May 17, 2012 10.1016/S0140-6736(12)60312-2 PMC3419820 22607825

[6] Vickers KC Palmisano BT Shoucri BM Shamburek RD Remaley AT MicroRNAs are transported in plasma and delivered to recipient cells by high-density lipoproteins. Nat Cell Biol. 2011;13:423-433 21423178 10.1038/ncb2210 PMC3074610

[7] Khera AV Cuchel M de la Llera-Moya M Rodrigues A Burke MF Jafri K French BC Phillips JA Mucksavage ML Wilensky RL Mohler ER Rothblat GH Rader DJ Cholesterol efflux capacity, high-density lipoprotein function, and atherosclerosis. N Engl J Med. 2011;364:127-135 21226578 10.1056/NEJMoa1001689 PMC3030449

[8] Besler C Heinrich H Rohrer L Doerries C Riwanto M Shih DM Chroni A Yonekawa K Stein S Schaefer N Mueller M Akhmedov A Daniil G Manes C Templin C Wyss C Maier W Tanner FC Matter CM Corti R Furlong C Lusis AJ von Eckardstein A Fogelman AM Lüscher TF Landmesser U Mechanisms underlying adverse effects of HDL on ENOS-activating pathways in patients with coronary artery disease. J Clin Invest. 2011;121:2693-2708 21701070 10.1172/JCI42946 PMC3223817

[9] Sorrentino SA Besler C Rohrer L Meyer M Heinrich K Bahlmann F Müller M Horvath T Doerries C Heinemann M Flemmer S Markowski A Manes C Bahr MJ Haller H Von Eckardstein A Drexler H Landmesser U Endothelial-vasoprotective effects of high-density lipoprotein are impaired in patients with type 2 diabetes mellitus but are improved after extended-release niacin therapy. Circulation. 2010;121:110-122 20026785 10.1161/CIRCULATIONAHA.108.836346

[10] Turner S Voogt J Davidson M Glass A Killion S Decaris J Mohammed H Minehira K Boban D Murphy E Luchoomun J Awada M Neese R Hellerstein M Measurement of reverse cholesterol transport pathways in humans: in vivo rates of free cholesterol efflux, esterification, and excretion. J Am Heart Assoc. 2012;1:e001826 doi: 10.1161/JAHA.112.001826 10.1161/JAHA.112.001826 PMC3487360 23130164

[11] Rosenson RS Brewer HB Jr Davidson WS Fayad ZA Fuster V Goldstein J Hellerstein M Jiang XC Phillips MC Rader DJ Remaley AT Rothblat GH Tall AR Yvan-Charvet L Cholesterol efflux and atheroprotection: advancing the concept of reverse cholesterol transport. Circulation. 2012;125:1905-1919 22508840 10.1161/CIRCULATIONAHA.111.066589 PMC4159082

[12] Glomset JA Wright JL Some properties of a cholesterol esterifying enzyme in human plasma. Biochim Biophys Acta. 1964;89:266-276 14205494 10.1016/0926-6569(64)90215-9

[13] Holleboom AG Kuivenhoven JA Vergeer M Hovingh GK van Miert JN Wareham NJ Kastelein JJ Khaw KT Boekholdt SM Plasma levels of lecithin:cholesterol acyltransferase and risk of future coronary artery disease in apparently healthy men and women: a prospective case-control analysis nested in the EPIC-Norfolk population study. J Lipid Res. 2010;51:416-421 19671930 10.1194/jlr.P900038-JLR200 PMC2803244

[14] Sethi AA Sampson M Warnick R Muniz N Vaisman B Nordestgaard BG Tybjaerg-Hansen A Remaley AT High pre-beta1 HDL concentrations and low lecithin:cholesterol acyltransferase activities are strong positive risk markers for ischemic heart disease and independent of HDL-cholesterol. Clin Chem. 2010;56:1128-1137 20511449 10.1373/clinchem.2009.139931 PMC4763716

[15] Tall AR Yvan-Charvet L Terasaka N Pagler T Wang N HDL, ABC transporters, and cholesterol efflux: implications for the treatment of atherosclerosis. Cell Metab. 2008;7:365-375 18460328 10.1016/j.cmet.2008.03.001

[16] Rayner KJ Esau CC Hussain FN McDaniel AL Marshall SM van Gils JM Ray TD Sheedy FJ Goedeke L Liu X Khatsenko OG Kaimal V Lees CJ Fernandez-Hernando C Fisher EA Temel RE Moore KJ Inhibition of miR-33a/b in non-human primates raises plasma HDL and lowers VLDL triglycerides. Nature. 2011;478:404-407 22012398 10.1038/nature10486 PMC3235584

[17] Singaraja RR Brunham LR Visscher H Kastelein JJ Hayden MR Efflux and atherosclerosis: the clinical and biochemical impact of variations in the ABCA1 gene. Arterioscler Thromb Vasc Biol. 2003;23:1322-1332 12763760 10.1161/01.ATV.0000078520.89539.77

[18] Patel DC Albrecht C Pavitt D Paul V Pourreyron C Newman SP Godsland IF Valabhji J Johnston DG Type 2 diabetes is associated with reduced ATP-binding cassette transporter A1 gene expression, protein and function. PLoS ONE. 2011;6:e22142 21829447 10.1371/journal.pone.0022142 PMC3144880

[19] Francone OL Fielding CJ Fielding PE Distribution of cell-derived cholesterol among plasma lipoproteins: a comparison of three techniques. J Lipid Res. 1990;31:2195-2200 2090713

[20] von Eckardstein A Huang Y Wu S Noseda G Assmann G Reverse cholesterol transport in plasma of patients with different forms of familial high density lipoprotein deficiency. Arterioscler Thromb Vasc Biol. 1995;15:690-701 10.1161/01.atv.15.5.691 7749883

[21] Huang Y von Eckardstein A Assmann G Cell-derived unesterified cholesterol recycles between low density lipoproteins and different high density lipoproteins for its effective esterification. Arterioscler Thromb. 1993;13:445-458 8443149 10.1161/01.atv.13.3.445

[22] Hoang A Drew BG Low H Remaley AT Nestel P Kingwell BA Sviridov D Mechanism of cholesterol efflux in humans after infusion of reconstituted high-density lipoprotein. Eur Heart J. 2012;33:657-665 21498847 10.1093/eurheartj/ehr103 PMC6590868

[23] Miida T Kawano M Fielding CJ Fielding PE Regulation of the concentration of pre beta high-density lipoprotein in normal plasma by cell membranes and lecithin-cholesterol acyltransferase activity. Biochemistry. 1992;31:11112-11117 1445850 10.1021/bi00160a022

[24] Kunitake ST Chen GC Kung SF Schilling JW Hardman DA Kane JP Pre-beta high density lipoprotein: unique disposition of apolipoprotein A-I increases susceptibility to proteolysis. Arteriosclerosis. 1990;10:25-30 2136993 10.1161/01.atv.10.1.25

[25] Haghpassand M Bourassa PA Francone OL Aiello RJ Monocyte/macrophage expression of ABCA1 has minimal contribution to plasma HDL levels. J Clin Invest. 2001;108:1315-1320 11696576 10.1172/JCI12810 PMC209438

[26] Brunham LR Singaraja RR Duong M Timmins JM Fievet C Bissada N Kang MH Samra A Fruchart JC McManus B Staels B Parks JS Hayden MR Tissue-specific roles of ABCA1 influence susceptibility to atherosclerosis. Arterioscler Thromb Vasc Biol. 2009;29:548-554 19201688 10.1161/ATVBAHA.108.182303

[27] Chan DC Hoang A Barrett PH Wong AT Nestel PJ Sviridov D Watts GF Apolipoprotein B-100 and ApoA-II kinetics as determinants of cellular cholesterol efflux. J Clin Endocrinol Metab Published online before print June 28, 2012 10.1210/jc.2012-1522 22745238

[28] Dietschy JM Turley SD Control of cholesterol turnover in the mouse. J Biol Chem. 2002;277:3801-3804 11733542 10.1074/jbc.R100057200

[29] Rothblat GH Phillips MC High-density lipoprotein heterogeneity and function in reverse cholesterol transport. Curr Opin Lipidol. 2010;21:229-238 20480549 10.1097/mol.0b013e328338472d PMC3215082

[30] von Eckardstein A Rohrer L Transendothelial lipoprotein transport and regulation of endothelial permeability and integrity by lipoproteins. Curr Opin Lipidol. 2009;20:197-205 19395962 10.1097/MOL.0b013e32832afd63

[31] Vergeer M Korporaal SJ Franssen R Meurs I Out R Hovingh GK Hoekstra M Sierts JA Dallinga-Thie GM Motazacker MM Holleboom AG Van Berkel TJ Kastelein JJ Van Eck M Kuivenhoven JA Genetic variant of the scavenger receptor BI in humans. N Engl J Med. 2011;364:136-145 21226579 10.1056/NEJMoa0907687

[32] Fabre AC Malaval C Ben Addi A Verdier C Pons V Serhan N Lichtenstein L Combes G Huby T Briand F Collet X Nijstad N Tietge UJ Robaye B Perret B Boeynaems JM Martinez LO P2y13 receptor is critical for reverse cholesterol transport. Hepatology. 2010;52:1477-1483 20830789 10.1002/hep.23897

[33] Brufau G Groen AK Kuipers F Reverse cholesterol transport revisited: contribution of biliary versus intestinal cholesterol excretion. Arterioscler Thromb Vasc Biol. 2011;31:1726-1733 21571685 10.1161/ATVBAHA.108.181206

